# Factors facilitating effective collaboration between labor and social security attorneys and medical professionals in supporting cancer survivors balancing treatment and work

**DOI:** 10.1016/j.apjon.2025.100732

**Published:** 2025-05-27

**Authors:** Sono Sugitani, Shinobu Yamada

**Affiliations:** Wakayama Medical University Graduate School of Health and Nursing Science, Wakayama, Japan

**Keywords:** Labor and social security attorneys, Medical professionals, Cancer survivors and balance treatment and work

## Abstract

**Objective:**

This study aims to identify the key factors that enable effective collaboration between labor and social security attorneys and health care professionals in supporting cancer survivors who are balancing treatment and work. By understanding what attorneys consider essential for successful collaboration with health care professionals, this research seeks to improve the interprofessional support system for cancer survivors managing both their health and work.

**Methods:**

A questionnaire survey was conducted among labor and Social Security Attorneys in two prefectures in Japan's Kansai region. Qualitative text analysis and hierarchical cluster analysis were used to identify essential factors for smooth coordination between these professionals and medical practitioners.

**Results:**

Twelve key factors were identified, including the need for clear medical information without reliance on internet searches, prognosis-based employment considerations, improved understanding of employment regulations by medical professionals, and enhanced information-sharing practices. Additionally, issues such as differences in perception between medical professionals and cancer survivors, the necessity of specifying work restrictions based on treatment policies, and the importance of estimated return-to-work timelines were highlighted.

**Conclusions:**

Effective collaboration between Labor and Social Security Attorneys and medical professionals requires clear, structured communication of medical information tailored to individual work conditions. Medical professionals should ensure that their recommendations regarding employment are specific, practical, and easily interpretable by non-medical professionals. Strengthening multidisciplinary cooperation will contribute to better support systems for cancer survivors in maintaining their employment while undergoing treatment.

## Introduction

Studies have shown that the number of cancer patients under the age of 50 is increasing rapidly worldwide.^1^ In japan about one in three cancer patients are in their 20s–60s, and many of them are working and outpatient treatment.^2^ In addition, 19.8% of employed people retired or quit their jobs after being diagnosed with cancer, and 56.8% retired or closed their jobs before the first treatment.^3^ Major survey results on the employment status of cancer patients in Japan show that the percentage of those working full-time decreased from 60% to 49%, the percentage of those working contract, part-time, or no work increased from 25.1% to 36.5%, compared to the percentage at the time of diagnosis. About one-fourth of those who were working at the time of diagnosis had lost their jobs at the time of the survey; about half of them requested to resign or be transferred voluntarily, 40% of them were instructed to do so by their companies. 55.2% continued to work in the same department at the same workplace after diagnosis.^4^ In a survey on the actual status of support for working cancer patients by nurses and the difficulties they face, “coordination of decisions on whether or not to work with employers” and “information sharing with employers” were almost not implemented. Therefore, supporting cancer survivors in balancing treatment and work has become an important issue in Japan.

In Japan, since the second Basic Plan to Promote Cancer Control Programs^5^ includes directions regarding work support for cancer patients and their family, various initiatives have been promoted. In addition, in 2013, as a part of comprehensive work support program for cancer patients, provision of consultation services by Labor and Social Security Attorneys began on a trial basis at cancer consultation and support centers in prefectural core cancer care hospitals and regional core cancer care hospitals which handle high-quality cancer treatment.

In Japan, there are Labor and social security attorney who provide day-to-day consultation in the field of labor issues. They are recipients of the Ministry of Health, Labor and Welfare who specialize in laws related to employee labor and social insurance, as well as personnel and labor management. They are closely involved in labor issues at production sites and can provide operational improvements and consulting services. The role of a labor and social security attorney is to stand between the parties involved and provide advice from a neutral standpoint in order to prevent labor problems from occurring. A major difference between a labor and social security attorney's role and a lawyer's is that only the lawyer is authorized to negotiate on behalf of the parties in the event of a problem. At the consultation support center of a cancer treatment base hospital, a labor and social security attorney provides consultation services to patients attending or hospitalized at the hospital. They are enrolled as a corporate advisor provides consultation services to cancer survivors who visit the hospital individually. Examples of consultations with the they are included: “The company does not offer me unpaid leave even though I am scheduled to undergo surgery,” “I am not allowed to take a long leave of absence because I am not a full-time employee,” and “I am worried that my colleagues will not understand about my cancer treatment.^6^ In this way, the role of they are involves assisting cancer survivors seeking to balance treatment and work is to provide practical advice on how to use the company's systems, how to consult with a person in charge in the workplace, and how to use social security systems .

In order to promote support for cancer survivors seeking to balance treatment and work, coordination between Labor and Social Security Attorneys and medical professionals is considered important. In general, coordination is defined as the process involving the mutual relationship among multiple individuals and organizations with a shared goal, where they build a cooperative relationship of their own initiative in order to address issues that cannot be resolved by each party individually and work together to achieve the goal.^7^ Within this coordination, how the patient is perceived and the amount of knowledge between persons in different professions varies greatly, this leads to differences in what is considered important. As a result, the contents of information provided do not always match with the required information, leading to problems in information sharing.^8^ In experiences gained from activities as a coordinator assisting patients seeking to balance treatment and work, the author had the opportunity to hear opinions from Labor and Social Security Attorneys involved in the support for cancer survivors seeking to balance treatment and work, such as “the documented opinion of the attending physician is difficult to understand because it includes abstract descriptions” and “what a worker (cancer survivor) said and the contents of documented opinion of the attending physician differ in some cases.” In order for health care professionals to smoothly collaborate with them in the support of cancer survivors seeking to balance treatment and work, understanding what they need to support cancer survivors is important.

The purpose of this study is to elucidate the factors that facilitate collaboration between labor and social security attorneys and health care professionals in supporting cancer survivors to balance their treatment and work. These factors include practical methods for information sharing, mutual understanding of roles among the professionals, and the quality of communication. By clarifying the factors that labor and social security attorneys deem necessary for effective collaboration with health care professionals, this research anticipates enhancing the interprofessional support system for cancer survivors seeking to manage both their treatment and careers.

## Methods

### Terms definitions

Cancer survivor: Irrespective of his/her health condition or response to treatment, a patient diagnosed with cancer and lives as a cancer patient from the time of diagnosis until the end of his/her life.^9^

Balancing treatment and work: Efforts to ensure that a patient does not miss opportunities to receive cancer treatment due to work, is not prevented from continuing to work due to the need for cancer treatment, and can continue to work with peace of mind while receiving the appropriate treatment.^10^

Effective collaboration: Activities by Labor and Social Security Attorneys and medical professionals to work together to promote support for cancer survivors seeking to balance treatment and work.^7^

### Subjects

Respondents to a questionnaire sent to 2121 Labor and Social Security Attorneys whose contact information is publicly available on the Japan Federation of Labor and Social Security Attorney's Associations website in six prefectures within the Kansai region. The purpose of the study, confidentiality, anonymity, and the voluntary nature of participation were fully explained in writing to Labor and Social Security Attorneys, their consent was obtained to participate in the survey.

### Data collection

**Fig. 1:**
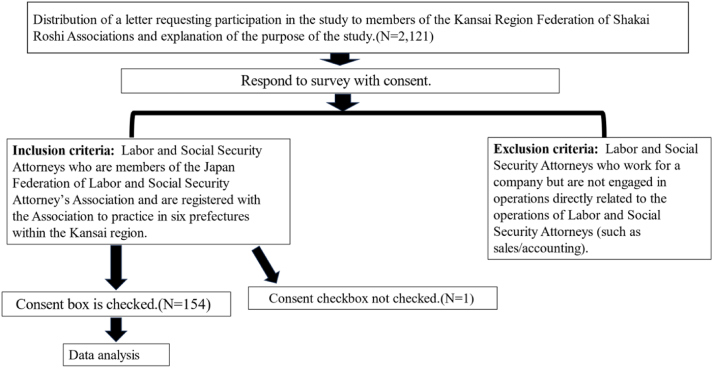
Flow of data collection.

This study was conducted from May 2023 to the end of August 2023. Inclusion criteria: Labor and Social Security Attorneys who are members of the Japan Federation of their Association and are registered with the Association to practice in six prefectures within the Kansai region (Fig. 1). Exclusion criteria: Labor and Social Security Attorneys who work for a company but are not engaged in operations directly related to the operations of Labor and Social Security Attorneys (such as sales/accounting). The labor and social security attorney will work to support cancer survivors in their workplaces and treatment hospitals. Therefore, the presence or absence of activities at hospitals shall not be included in the exclusion criteria (Fig. 1).

### Study method

Inquiry forms with a QR code were sent by postal mail to representatives of offices registered in the Japan Federation of Labor and Social Security Attorney's Association in each of the six prefectures in the Kansai region to request participation in the study. To reply to the questionnaire, use of either an Internet-based response form using a Google form or completion of the questionnaire sheet sent by mail was possible. Survey items included a total of ten questions regarding information needed before and during treatment, matters which require information sharing, and questions regarding the attributes of the Labor and Social Security Attorneys. Free description type responses and multiple-choice responses were included.

Regarding basic attributes, gender, age, years of experience, main field of activities (private firm, company consultant), location of workplace, main operations, and experience in assisting cancer survivors seeking to balance treatment and work were considered. Ten questions (Table 1) were prepared based on various guidelines and other information^11^, ^12^, ^13^, ^14^, ^15^, ^16^ to assist cancer survivors seeking to balance treatment and work in the workplace. The analysis method used was econometric text analysis and hierarchical cluster analysis.

**Table 1:** Question item.

1. In a workplace where a cancer survivor who has not yet received treatment works, what medical information is needed to address the work style of the cancer survivor according to his/her health condition?
2. When the day off or leave of absence system of an office is considered or reviewed, or introduction of a new system is being considered before treatment begins, what medical information is required?
3. In a workplace where a cancer survivor who is receiving outpatient treatment works, what medical information is needed to address the work style of the cancer survivor according to his/her health condition?
4. When the day off or leave of absence system of an office is considered or reviewed, or introduction of a new system is being considered during the outpatient treatment, what medical information is required?
5. When a labor and social security attorney is assisting a cancer survivor to balance treatment and work, what information does he/she want medical professionals to know?
6. When information is shared, what do you want medical professionals to consider?
7. What medical expressions and terms that are difficult to understand?
8. If there are any medical expressions and terms that are difficult to understand, what sources of information collection are used for confirmation?
9. Regarding the importance of information sharing between medical institutions and labor and social security attorney in order to support cancer survivors in the workplace, what should be considered?
10. Regarding the importance of information sharing to support cancer survivors seeking to balance treatment and work, what things should a labor and social security attorney keep in mind?

### Data analysis

Text data was first created from the responses obtained from Labor and Social Security Attorneys. We loaded the text data into the free text mining software KH Coder (Ver.3),^17^ converted the text data into a form that could be quantitatively analyzed, performed morphological analysis to classify sentences into parts of speech such as nouns and verbs. In addition, synonyms were processed so that span would be interpreted as duration, chemo as chemotherapy and net as internet, for example. The frequency of occurrence of extracted terms was calculated to grasp outline of the data. By confirming the frequency of occurrence of extracted terms, we can verify whether terms that are important for analysis are not divided unnaturally and whether the occurrence frequency of terms that are considered unrelated to the analysis is not high.^18^^,^^19^

We then performed a hierarchical cluster analysis to help cancer survivors structure their descriptions of what Labor and Social Security Attorneys looks for in a health care provider in order to continue balancing treatment and work. The merger level was used as a reference for determining the number of clusters, and clusters were created by the Ward method and the Jaccard coefficient. The Merger level is the degree of dissimilarity (degree of dissimilarity), and by plotting this value, the optimal number of clusters can be determined. A sharp rise in the level of consolidation is an indication of the number of clusters.^21^^,^^20^ The Ward method is a balanced method among cluster analysis methods because it takes into account the minimization of the sum of data squares in clusters.^20^ The Jaccard coefficient used as an index of similarity indicates the proportion of sentences in which both words co-occur in sentences in which either word A or word B appears.^18^, ^20^, ^21^, ^22^

**Fig. 2:**
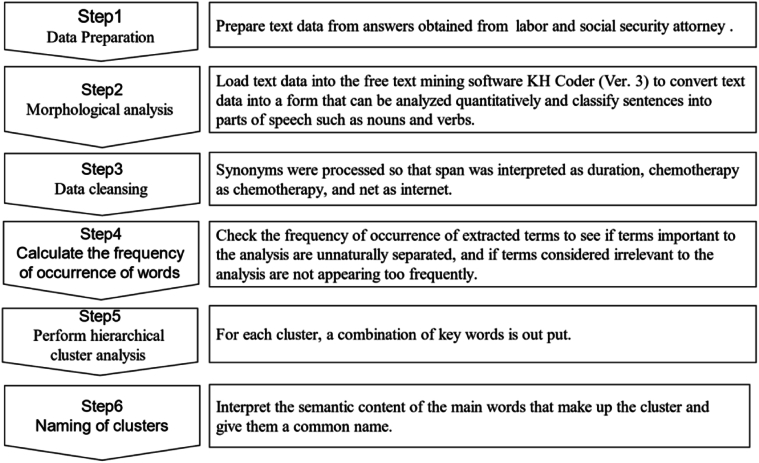
Data analysis flow.

The clusters were named using the Key Words in Context (KWIC) concordance, and we checked the original text and how the extracted words were used from the surrounding context (Fig. 2). The clusters were named using the Key Words in Context (KWIC) concordance, and we checked the original text and how the extracted words were used from the surrounding context. In the entire analysis process, from data processing of the questionnaire survey to the naming of clusters, efforts were made to ensure reliability and validity through repeated discussions with the research supervisor, who has practical experience as a nurse specialist in oncology nursing and has experience in text mining.

## Results

Inquiry forms were sent to 2121 Labor and Social Security Attorneys, and 155 responses were received, resulting in a response rate of 7.3% (155/2121). Among the returned forms, one subject did not provide consent to participate in the study and was excluded from further analysis. Consequently, the remaining 154 responses were considered valid for the analysis set, representing 99.4% of the received responses (154/155).

Regarding the attributes of the 154 study participants, the largest age group was individuals in their forties (28.6%, *n* ​= ​44), followed by those in their fifties (27.3%, *n* ​= ​42) and sixties (27.3%, *n* ​= ​42). The mean age of the participants was 55.6 years. The mean years of experience as a Labor and Social Security Attorney was 14.4 years. Sixty-four participants (41.6%) reported having experience assisting cancer survivors seeking to balance treatment and work, while 90 participants (58.4%) reported having no such experience (Table 2).

**Table 2:** Subject attributes.

		*n*	%

Age (years)	∼39	7	4.5
	40∼49	45	29.5
	50∼59	42	27.2
	60∼69	42	27.2
	70∼79	15	9.7
	80∼89	3	1.9
Years of experience as a labor and social security attorney	1∼4	19	12.3
	5∼9	27	17.5
	10∼14	35	22.7
	15∼19	21	13.6
	20∼24	33	21.4
	25∼29	10	6.4
	30∼	9	5.8
Experience assisting cancer survivors seeking to balance treatment and work	Yes	64	41.5
	No	90	58.4

Extracted terms subject to analysis: Terms with a minimum frequency of occurrence of 20 were included in the analysis, and 92 terms were generated (Table 3).

**Table 3:** Extracted terms subject to analysis.

Extracted term	Occurrence frequency	Extracted term	Occurrence frequency	Extracted term	Occurrence frequency
Treatment	418	Day off	48	Burden	26
Contents	191	Understanding	48	Policy	26
Necessary	185	To do work	46	Rough estimate	26
Duration	183	Plan	43	Search	25
Work	165	Response	43	Outlook	25
The person	145	Listen to	43	Personal information	25
Acceptable	138	Investigate	42	Timing	25
Time	138	Judgement	41	Sharing	24
Operation	124	Condition	40	Office	24
Extent	119	Planned	40	Employee	24
Consideration	105	Impact	39	Technical terms	24
Patient	100	Symptoms	39	Many	24
Company	99	Know	39	Communicate	24
Physician	76	Return to work	38	Number of days	24
System	69	Explanation	36	Hospitalization	24
Internet	68	Presence or absence	36	Scope	24
Workplace	68	Enterprise	35	Return	24
Outpatient visit	66	Medical certificate	35	Understand	24
Circumstances	65	Restriction	35	Required	24
Think of	64	Physical condition	34	Potential	23
Confirmation	61	Disease condition	34	Change	23
Working	60	Frequency	33	Desire	22
Adverse reaction	60	Estimation	32	Leave	22
Medical institution	59	Injury and sickness allowance	32	Continuation	22
Future	59	Disease name	32	Capable	22
Concrete	57	Attending physician	31	Disease	22
Labor	57	Information sharing	30	Position	22
Medical practice	54	Work duties	30	Myself	21
Leave of absence	53	How to	30	Opinion	20
Employment	51	Current	26	Expected	20
Be working	51	Directly	26		

**Fig. 3:**
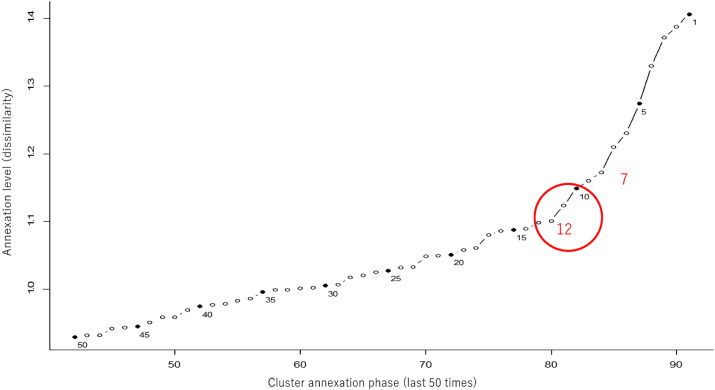
Merger level. The vertical axis is annexation level (dissimilarity), and the horizontal axis is cluster annexation phase (last 50 times). Numeric labels in the plot are the total number of clusters after annexation.

The highest dissimilarity at the merger level was 7. However, with this number, valuable terms extracted may be overlooked. Therefore, we set 12 as the number of clusters with the second highest degree of difference (Fig. 3).

**Fig. 4:**
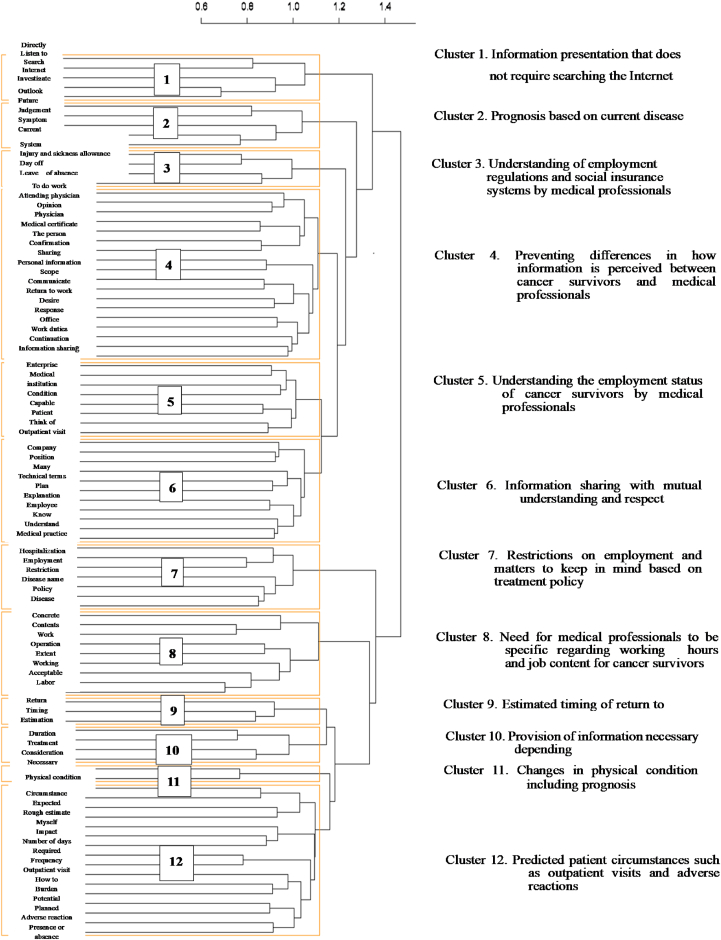
Dendrogram based on hierarchical cluster analysis, and cluster names. Extracted words are shown on the left side and on the right side, the name of each cluster is shown.

As a result, the dendrogram shown in Table 1 was generated and terms were divided into 12 clusters (Fig. 4). The number of extracted terms is set in parentheses (), representative responses are set in quotation marks “” and the name of the cluster in brackets []. The number of extracted terms is set in parentheses (), representative responses are set in quotation marks “” and the name of the cluster in brackets [].

Moreover, Cluster names are named by confirming how the extracted words are used in the original responses of Labor and Social Security Attorneys (Table 4).

**Table 4:** Extracted terms, number of terms, and representative responses.

Cluster	Extracted terms	Number of terms	Representative responses

1.Information presentation that does not require searching the Internet.	Internet Listen to investigate Directly Search	68 43 42 26 25	Technical terms are still difficult to understand. Now, we have to obtain information from generative AI or search for the definition of the terms using the Internet. However, I don't know if the information obtained from these sources or if the intention of the terms based on such information is correct. I Have searched for the terms over the Internet, but eventually, I had to directly ask the physician what needs to be done.
2. Prognosis based on current disease condition.	Future Judgement Disease condition Current Outlook	59 41 34 26 25	Outlook of the current situation and the future. Outlook for future work and future treatment plan, current situation, current disease condition, frequency of outpatient visits, the degree of heavy work that is acceptable or not acceptable to the person.
3. Understanding of employment regulations and social insurance systems by medical professionals	System Leave of absence Day off Injury and sickness allowance	69 53 48 32	I want medical professionals to know about the leave of absence system, rules on employment, and the need to cooperate when returning application forms to a patient when he/she applies for injury and sickness allowance or disability pension. I want medical professionals to know that some diseases are specifically mentioned for the leave of absence, injury and sickness allowance, or disability pension systems.
4. Preventing differences in how information is perceived between cancer survivors and medical professionals.	The person Physician Confirmation To do work Response Return to work Medical certificate Attending physician Work duties Information sharing Personal information Office Communicate Sharing Scope Continuation Desire Opinion	145 76 61 46 43 38 35 31 30 30 30 24 24 24 24 22 21 20	Difference between the wishes of the person and the judgment of the physician. I want physicians to concretely understand as far as possible that desires of the person and there is a gap between what a company can do which greatly varies dues to differences between individual companies and their limits, and in particular, what kind of work the person is doing. It is important correctly convey to the company the extent a person can work because the person is likely to overextend him/herself. Since information may be change when passed down from the patient correct information should be communicated.

Cluster	Extracted terms	Number of terms	Representative responses

5. Understanding of employment status of cancer survivors by medical professionals.	Patient Workplace Think of medical institution Be working Condition Company Capable	100 68 64 59 51 41 35 20	Because length of duration of leave of absence tends to be proportional to company scale, I think information regarding predicted treatment duration is very important, even for other employees who work in place of the patient, especially in small businesses. The wishes of the person and those of the company greatly vary depending on the individual company and what the enterprise can do is limited. In particular, wishes of a person are less likely to be accepted by a small and medium-sized company. Although employees are the most important, creating opportunities to discuss practical responses depending on the size of the business, number of employees, and business conditions should be considered.
6. Information sharing with mutual understanding and respect.	Company Medical practice Understanding Plan Know Explanation Employee Many Technical terms Position Understand	99 54 48 43 39 36 24 24 24 22 22	I Think patients can understand more easily if technical terms are rephrased with easy-to-understand words when possible. When cancer patients consult with a specialist, the specialist may be unable to provide good advice if the patient does not fully understand their disease or the treatment plan. While it is quite normal for a company not to have medical knowledge, but neither do many labor and social security attorney. Therefore, as medical practice continues to advance day by day, it is helpful if medical professionals could express themselves without using technical terms so that treatment plan, etc. can be easily understood
7. Restrictions on employment and matters to keep in mind based on treatment policy.	Employment Symptoms Restriction Disease name Policy Hospitalization Disease	51 39 35 32 26 24 22	Employment restrictions and matters to keep in mind. Expressions that can be understood even by persons who are not involved in medical practice should be used. Disease name and treatment details: Not a description of the actual treatment but medical information on conditions that affect the way the patient lives and daily or weekly restrictions, such as what kind of work would result in worsening symptoms. Hospitalization, work restrictions, estimated time to return to work and conditions for return to work, status of cancer, and cancer treatment policy.
8. Need for medical professional to be specific regarding working hours and job content for cancer survivors.	Contents Work Time Acceptable Operation Extent Working Labor Concrete	191 165 138 124 119 60 60 57 57	I want medical professionals to correctly understand the contents of the work of the person and determine the extent to which the person can perform such work. Scope of acceptable work according to the business contents of the company. Descriptions in the medical certificate such as “light work” are so vague that the office cannot determine the proper work. Labor and social security attorney consider how the patient's work and life are restricted due to the disease, and I want the medical professionals to be specific regarding the contents of diagnosis. If light work is determined as acceptable for the patient, it is very difficult to interpret “light work” in the labor management setting.

Cluster	Extracted terms	Number of terms	Representative responses
9. Estimated timing of return to work.	Estimation Timing Return	32 25 24	Information about estimated timing of return to work as the basis to determine the need for days off or leave of absence. Estimated duration of treatment. How much time (days off) is needed for treatment, how long will treatment be, and when can the worker be expected to return to work. Consideration required after return to work, can the person do the same work after treatment as that before treatment.
10. Provision of information necessary depending on treatment duration.	Treatment Necessary Duration Consideration	418 185 183 105	How physical condition will change at what stage of treatment, matters to consider, contents of unacceptable work, acceptable work hours, needed work environment, contents of acceptable work, and acceptable number of days of work. The company needs information regarding treatment policy, treatment duration, and estimated time of recovery from the physical burden rather than technical medical information. Matters to consider during work: Is a stoma in place, condition of excretion, eating method, and physical mobility.
11. Changes in physical condition including prognosis.	Physical condition Change	34 23	Information should not be provided through the filter of the patient but accurate information on the patient's current condition. For example, information that is conducive to understanding of what changes in physical condition will occur over the process of treatment, whether sudden poor physical condition will occur, and to what extent recovery, as compared to before disease onset, can be expected. I want medical professionals to describe points to keep in mind while understanding that labor and social security attorney are not familiar with the changes in physical condition associated with the treatment or its effects.
12. Predicted patient circumstances such as outpatient visits and adverse reactions	Outpatient visit Circumstances Adverse reaction Planned Impact Presence or absence Frequency How to rough estimate Burden Number of days Required Potential Leave Myself Expected	66 65 60 40 39 36 33 30 26 26 24 24 23 22 21 20	Outpatient visits planned during treatment, time required per outpatient visit, potential adverse reactions, number of days of leave needed, details of changes in physical condition, and how to rest. Effect of disease condition on work. Frequency of outpatient visits. What are the adverse reactions. Frequency and length required for each outpatient visit for examination, etc., treatment duration and physical effect of the treatment that can be presently expected Whether work is possible according to work duties, things to consider to reduce burden, and information about outpatient treatment. Information regarding expected circumstances associated with treatment is needed. If a severe adverse reaction occurs during outpatient treatment, even going to office may be difficult.

Cluster 1: Includes five extracted terms, such as “the Internet”, and “listen to”, and the following responses: “Technical terms are still difficult to understand. Now, we have to obtain information from generative AI or search for the definition of the terms using the Internet. However, I don't know if the information obtained from these sources or if the intention of the terms based on such information is correct. Discrepancy between parties could be reduced if a straightforward and detailed explanation could be provided.” This cluster was named [Information presentation that does not require searching the Internet.]

Cluster 2: Includes five terms, such as “future”, and “judgment”, and the following responses: “Materials used to judge future matters such as estimated time of return to work, and whether days off or a leave of absence is required. How heavy is the burden on the worker due to current disease condition and current work load?” The cluster was named [Prognosis based on current disease condition.]

Cluster 3: Includes four terms, such as “system”, and “leave of absence”, and the following responses: “I want medical professionals to know about the leave of absence system, rules on employment, and the need to cooperate when returning application forms to a patient when he/she applies for injury and sickness allowance or disability pension.” This cluster was named [Understanding employment regulations and social insurance systems by medical professionals.]

Cluster 4: Includes 18 terms, such as “the person”, and “physician”, and the following responses: “Information received from the cancer survivor and information from medical professionals seems to differ depending on the perception of the cancer survivor. The cluster was named [Preventing differences in how information is perceived between cancer survivors and medical professionals.]

Cluster 5: Includes 8 terms such as “patient”, and “workplace”, and the following responses: “Since it is difficult to flexibly respond in small and medium-sized enterprises, considering a workplace environment where the patient can work is desirable.” The cluster was named [Understanding the employment status of cancer survivors by medical professionals.].

Cluster 6: Includes 11 terms such as “company”, and “medical practice”, and the following responses: “Medical professionals and medical institutions do not know enough about Labor and Social Security Attorneys. I want them to understand the work of Labor and Social Security Attorneys.” The cluster was named [Information sharing with mutual understanding and respect.]

Cluster 7: Includes 7 terms such as “employment”, and “symptoms”, and the following responses: “Disease name and treatment details: Not a description of the actual treatment but medical information on conditions that affect the way the patient lives and daily or weekly restrictions, such as what kind of work would result in worsening symptoms.” The cluster was named [Restrictions on employment and matters to keep in mind based on treatment policy.]

Cluster 8: Includes 9 terms such as “contents”, and “work”, and the following responses: “Labor and Social Security Attorneys consider how the patient's work and life are restricted due to the disease, and I want the medical professionals to be specific regarding the contents of diagnosis. If light work is determined as acceptable for the patient, it is very difficult to interpret “light work” in the labor management setting.” The cluster was named [Need for medical professionals to be specific regarding working hours and job content for cancer survivors.]

Cluster 9: Includes 3 terms, “estimation”, and “timing”, and the following responses: “Estimated treatment duration: Explanation on how much time (days off) is needed for treatment, how long will treatment be, and when can the worker be expected to return to work. A description of the estimated time of return.” The cluster was named [Estimated timing of return to work.]

Cluster 10: Includes 4 terms such as “treatment”, and “necessary”, and the following responses: “How physical condition will change at what stage of treatment, matters to consider, contents of unacceptable work, acceptable work hours, needed work environment, contents of acceptable work, and acceptable number of days of work.” The cluster was named [Provision of information necessary depending on treatment duration.]

Cluster 11: Includes 2 terms, “physical condition” and “change”, and the following responses: “I want medical professionals to describe points to keep in mind while understanding that Labor and Social Security Attorneys are not familiar with the changes in physical condition associated with the treatment or its effects.” The cluster was named [Expected changes in patient condition including prognosis.].

Cluster 12: Includes 16 terms, such as “outpatient visit”, and “circumstances”, and the following responses: “Information regarding expected circumstances associated with treatment is needed. If a severe adverse reaction occurs during outpatient treatment, even going to office may be difficult.” The cluster was named [Predicted patient circumstances such as outpatient visits and adverse reactions.]

## Discussion

### Analytical methods

In this study, we used quantitative text analysis and hierarchical cluster analysis to structure the open-ended answers obtained from the Labor and Social Security Attorneys. And clarified what their looking for in medical professionals when supporting cancer survivors in balancing work and treatment. Quantitative text analysis uses multivariate analysis to organize and analyze text-type data, qualitatively analyze its contents, and interpret the results.^23^ Qualitative research is an important research method that deeply explores the inner experiences of subjects, but it is difficult to process a large amount of data at once. In addition, it is difficult to maintain reproducibility and credibility as a limitation of qualitative research.^24^ In recent years, mixed research using both research methods have been introduced, one of which is quantitative text analysis.^21^ Quantitative text analysis has the advantage of eliminating manual work in summarizing and presenting text data such as responses through multivariate analysis, eliminating bias due to the analyst's theory and awareness of problems, and ensuring the objectivity, reliability, and transparency of the analysis.^20^^,^^23^, ^24^, ^25^, ^26^ In addition, hierarchical cluster analysis can collect and classify similar open-ended responses with different elements and tendencies.^23^ By analyzing unstructured texts qualitative psychology of research subjects can be obtained. Qualitative means that it cannot be put into numbers. Quantitative data is numerical data, can be seen as a result of the behavior of the research subjects. But we thought that it is necessary to conduct qualitative analysis such as questionnaires to determine the reasons why it is necessary. In this study, we thougunt that the hierarchical cluster analysis allowed us to classify a similar large of free responses with different elements and tendencies obtained from labor and social security attorney.

In addition, this was a mixed study using text mining, with the primary goal of gaining deeper insights from the qualitative data. Quantitative analysis was conducted to augment the results of the qualitative study and increase its reliability. In qualitative research, data richness, diversity, depth of analysis are more important than quantitative sample size, and in this study, 154 responses were obtained. Generally, 154 responses are considered a sufficient sample size for qualitative research.^27^ We believe that even with this number of responses, a deep analysis is possible because the nature of the text mining methodology allows rich information to be extracted from individual responses.

### Presentation of medical information

Cluster 1 [Information presentation that does not require searching the Internet], Cluster 2 [Prognosis based on current disease condition], Cluster 7 [Restrictions on employment and matters to keep in mind based on treatment policy], Cluster 8 [Need for medical professionals to be specific regarding working hours and job content for cancer survivors], Cluster 9 [Estimated timing of return to work], Cluster 10 [Provision of information necessary depending on treatment duration], Cluster 11 [Changes in physical condition including prognosis], and Cluster 12 [Predicted patient circumstances such as outpatient visits and adverse reactions.] are considered to be factors in the effective collaboration of medical information between Labor and Social Security Attorneys and medical professionals involved in the support of cancer survivors seeking to balance treatment and work. The guidelines by the Ministry of Health, Labor and Welfare^28^ include a form for patients to fill out information such as current symptoms, treatment schedule, whether they will continue to work after discharge or during treatment, and the nature of their work, which are considered important when seeking the doctor's opinion regarding the treatment status and continuing to work. There are many similarities between the contents of these items and the results of this study. These medical information items are considered fundamental when considering the support of cancer survivors seeking to balance treatment and work.

In a workplace where cancer survivors receiving treatment work, arrangements such as setting the time for a treatment appointment later in the day or taking a day off with pay must be made.^29^^,^^30^ In this respect, Labor and Social Security Attorneys must consider medical information regarding physical condition and treatment of the cancer survivor. Labor and Social Security Attorneys consider specific medical information regarding physical condition and treatment of cancer survivors necessary for cancer survivors to continue working.^31^ Medical professionals do provide much information to determine whether a cancer survivor can continue working, such as future treatment policy, time needed for treatment, effect of treatment on the physical condition of the patient, and obstacles to work in the early phase after diagnosis of cancer.^32^ However, considered to lead to vague information in documents including the opinion of the attending physician. Labor and Social Security Attorneys believe that there is a need for specific information to develop an employment plan in order to adjust the work environment of cancer survivors, to apply for social security systems such as leave of absence and sickness benefits, and to prepare for the improvement of the workplace environment in which cancer survivors work. For medical professionals including physicians, nurses, pharmacists, registered dietitians, social workers, and rehabilitation experts involved in the care of cancer survivors of working age, frequent communication regarding how to support cancer survivors seeking to balance treatment and work based on each professional's specialized knowledge is important. And presenting medical information in which the opinions and knowledge of each professional has been comprehensively summarized is considered important. Being available as well as a coordinator characterises the nurse's role across health care systems. As a measure to facilitate these efforts, we believe that it is important for nurses to play a role in connecting and coordinating between other professions. Nurses act as a link between different professions, between patients and families, contributing to ensuring the quality of care for individual patients. As an issue for the future, we believe that it will be important to educate nurses who can work to support the employment of cancer survivors. Therefore, we believe it will be important for oncology nursing practice to develop nurses who can work to support the employment of cancer survivors.

Regarding medical terms, rephrasing or an easy-to-understand explanation so that even non-specialists can accurately understand should be considered.^33^ For Labor and Social Security Attorneys, while terms related to labor acts or social security are used on a daily basis, medical terms are considered to be highly unusual. When medical professionals provide medical information via the documented opinion of an attending physician, etc., consideration of the differences between objective events that may hinder a cancer survivor from carrying out his/her duties and usual operational management in the office is needed. Therefore, medical professionals need to understand how the medical information will be used by Labor and Social Security Attorneys and keep in mind the difference in job contents between medical professionals and Labor and Social Security Attorneys. For instance, instead of using medical terms like 'courses' or 'cycles' for anticancer treatment schedules, explain them simply using ‘number of days,' ‘chemotherapy room visits,' ‘likely adverse reaction periods,' and guidance on balancing treatment and work."

### Health care professionals support cancer survivors

Cluster 3 [Understanding of employment regulations and social insurance systems by medical professionals], Cluster 4 [Preventing differences in how information is perceived between cancer survivors and medical professionals], Cluster 5 [Understanding the employment status of cancer survivors by medical professionals], and Cluster 6 [Information sharing with mutual understanding and respect] are considered relevant for information sharing between Labor and Social Security Attorneys and medical professionals.

Support for cancer survivors seeking to balance treatment and work starts with a request from the cancer survivor. Next, information is shared between the medical institution and the company office based on written documents or direct presentation of medical information from the cancer survivor.^34^^,^^35^ Among the representative responses from Labor and Social Security Attorneys regarding medical information received from the cancer survivor, ” Information received from the cancer survivor and information from medical professionals seems to differ depending on the perception of the cancer survivor.” From these responses, it is possible that cancer survivors do not communicate their medical conditions and treatments well at work or at work. As a result, it is possible that medical information from medical professionals has not been passed on to Labor and Social Security Attorneys.

A cancer survivor may be mentally preoccupied with accepting and coping with his/her disease; for example, he/she cannot stop thinking about his/her disease, adverse reactions, family concerns or dissatisfaction, etc., and as a result, may not have the strength to collect the necessary information.^36^^,^^37^ As a consequence, cancer survivors who are working may not be able to correctly take advantage of the medial information that is needed to balance treatment and work. And it can be difficult for Labor and Social Security Attorneys to determine how they should make sense of the medical information provided through cancer survivors. Health professionals should guide cancer survivors to effectively communicate their medical needs to Labor and Social Security Attorneys so that they can best coordinate their work. In order for health care professionals to give appropriate advice to cancer survivors, they need to spend enough time interviewing cancer survivors, understanding their employment status, and understanding the social insurance system in their workplace. Further, the occupations of cancer survivors widely vary and some cancer survivors may have a difficulty continuing in the same work as that before diagnosis of cancer due to adverse reactions or sequela due to the treatment. Cancer survivors may use their energy to accept and cope with their diseases; for example, they cannot get anxiety and dissatisfaction about adverse reactions or their family and things about the disease out of their mind, and as a result, they may have no remaining energy to collect necessary information.^33^, ^34^, ^35^ Thus, working cancer survivors may not have sufficient energy to correctly use medial information that helps balance treatment and work.

Therefore, when medical information is provided via cancer survivors, it is considered important that cancer survivors themselves correctly use information necessary to balance treatment and work to lead to presentation of medical information that Labor and social attorneys require. To realize this, medical professionals should continuously provide supports such as careful selection, correction, and supplementation of information required for cancer survivors to balance treatment and work according to their sense of values and healthy phase.

### Limitations

In this study, cancer survivors were tabulated regardless of whether they had experience in balancing treatment and work. Therefore, there may be a difference in the perception of support for balancing treatment and work for cancer survivors depending on the presence or absence of experience. In addition, since there are many duties of labor and social security attorneys, so they tend to speak in their own fields of expertise, it is possible that the answers may be biased.

The Kansai region exhibits a high concentration of core hospitals providing advanced cancer care, accounting for approximately 27%^38^ of the national total of cancer care cooperation hospitals. Additionally, the number of registered Labor and Social Security Attorneys in Kansai is about 20%^39^ of the national Fig., which is high relative to the population, suggesting potentially active support for utilizing the social security system. Given this context, the Kansai region is likely to offer insightful cases regarding multidisciplinary collaboration and the application of the social security system for cancer patients. However, we believe that this regional characteristic may affect the generality of the results for a survey limited to the Kansai region. In the future, we would like to conduct similar surveys in other regions and make comparisons among regions to obtain more universal findings. We believe that continuing this research and utilizing the data obtained will help further support cancer survivors who are trying to balance treatment and work.

## Conclusions

Twelve key factors were identified that support work-life balance and promote effective working styles among cancer survivors, as well as facilitate collaboration between Labor and Social Security Attorneys and medical professionals. These include the need for medical professionals to better understand employment regulations and to improve information-sharing practices. The study also highlighted challenges such as differing perceptions between medical professionals and cancer survivors, the need to clearly specify work restrictions based on treatment policies, and the importance of providing an estimated timeline for returning to work.

## CRediT authorship contribution statement

**SS**: Conceptualization, Methodology, Investigation, Formal analysis, Writing – Original Draft. **SY**: Supervision, Writing – Review & Editing. All authors have read and approved the final manuscript.

## Ethics statement

The study was approved by the Ethical Review Committee of Wakayama Medical University, Japan (Approval No. 3887) and was conducted in accordance with the 1964 Helsinki Declaration and its later amendments or comparable ethical standards. All participants provided written informed consent.

## Data availability

The data are not publicly available due to ethical restrictions. Data are available from the authors upon reasonable request and with permission of the Ethical Review Committee.

## Declaration of generative AI and AI-assisted technologies in the writing process

No AI tools/services were used during the preparation of this work.

## Funding

This study was funded by the Yasuda Memorial Medical Foundation Cancer Nursing Research Grant (Grant No. FY2022). The funders had no role in considering the study design or in the collection, analysis, interpretation of data, writing of the report, or decision to submit the article for publication.

## Declaration of competing interest

The authors declare no conflict of interest.
